# Prolonged Duration Topical Corneal Anesthesia With the Cationic Lidocaine Derivative QX-314

**DOI:** 10.1167/tvst.8.5.28

**Published:** 2019-10-17

**Authors:** Alan G. Woodruff, Claudia M. Santamaria, Manisha Mehta, Grant L. Pemberton, Kathleen Cullion, Daniel S. Kohane

**Affiliations:** 1 Kohane Lab for Biomaterials and Drug Delivery, Department of Anesthesia, Perioperative and Pain Medicine, Division of Critical Care, Boston Children's Hospital, Boston, MA, USA; 2 Harvard Medical School, Boston, MA, USA; 3 David H. Koch Institute, Massachusetts Institute of Technology, Cambridge, MA, USA; 4 Department of Medicine, Division of Medicine Critical Care, Boston Children's Hospital, Harvard Medical School, Boston, MA, USA

**Keywords:** cornea, drug toxicity/drug effect, trauma, wound healing, refractive surgery

## Abstract

**Purpose:**

Topical corneal local anesthetics are short acting and may impair corneal healing. In this study we compared corneal anesthesia and toxicity of topically applied *N*-ethyl lidocaine (QX-314) versus the conventional local anesthetic, proparacaine (PPC).

**Methods:**

Various concentrations of QX-314 and 15 mM (0.5%) PPC were topically applied to rat corneas. Corneal anesthesia was assessed with a Cochet-Bonnet esthesiometer at predetermined time points. PC12 cells were exposed to the same solutions to assess cytotoxicity. Repeated topical corneal administration in rats was then used to assess for histologic evidence of toxicity. Finally, we created uniform corneal epithelial defects in rats and assessed the effect of repeated administration of these compounds on the defect healing rate.

**Results:**

QX-314 (15 mM) and PPC (15 mM) caused similar total duration (114 ± 17 and 87 ± 16 minutes, respectively; *P* = 0.06) of anesthesia. The depth of anesthesia was similar between these low-dose groups at 15 minutes after application (1.8 ± 0.3- and 2.0 ± 0.8-cm filament lengths). QX-314 (100 mM) provided more prolonged corneal anesthesia (174 ± 13 minutes; *P* < 0.0001), with improved depth at 15 minutes (0.7 ± 0.3-cm filament length; *P* = 0.007). All tested concentrations of QX-314 demonstrated similar or less toxicity than 0.5% PPC.

**Conclusions:**

Topical administration of QX-314 is effective for corneal anesthesia and demonstrates no histologic signs of local toxicity in a rodent model. In higher concentrations, QX-314 provides more than twofold the duration of anesthetic effect than does 0.5% PPC.

**Translational Relevance:**

Our study reveals a clinically relevant compound providing prolonged duration topical corneal anesthesia.

## Introduction

The human cornea is densely innervated and exquisitely sensitive to noxious stimuli.^1^,^2^ As corneal, cataract, and refractive surgeries are now customarily performed without general anesthesia, ophthalmologists rely on topical or regional administration of amino-amide and amino-ester local anesthetics (LA) to facilitate painless surgery.^3^,^4^ Single-dose administration of such agents is also common in the acute care setting to facilitate fluorescein examination and irrigation of debris in the setting of corneal abrasions.^5^,^6^ Shortcomings of available topical LA agents include a short duration of effect,^7^ the potential for corneal toxicity,^7^,^8^ and the retardation of corneal epithelial healing.^9^,^10^ It is thus desirable to find alternative topical ophthalmic anesthetics with prolonged duration of effect and reduced local toxicity.

*N*-ethyl-lidocaine (QX-314) is a lidocaine derivative with a quaternary amine moiety conferring an obligate positive charge.^11^ Perineural injection of QX-314 provides prolonged duration local anesthesia to peripheral nerves.^12^,^13^ However, topical administration of QX-314 on the cornea has not been studied. Like most conventional LAs, QX-314 and similar molecules used for peripheral nerve block can cause myotoxicity and, at higher doses, neurotoxicity.^12^,^14^ However, neurotoxicity generally occurs at high concentrations, and there is no muscle on or in the cornea. Consequently, we have hypothesized that QX314 would provide prolonged duration corneal anesthesia after topical ophthalmic administration without local toxicity. We assess corneal local anesthesia with a Cochet-Bonnet esthesiometer. We assess neurotoxicity in vitro and tissue toxicity histologically and by investigating the effect of QX314 on corneal epithelial healing after corneal debridement.

## Materials and Methods

### Materials

QX-314 (Sigma Aldrich Corp., St. Louis, MO) was prepared at various concentrations in 0.9% sterile saline. Drug solutions were prepared within 2 hours of use. Proparacaine (PPC) hydrochloride ophthalmic solution 0.5% (pH 3.5–6; Akorn, Inc., Lake Forest, IL) was aliquoted for each use from a stock bottle stored at 4°C and was warmed to room temperature before use.

The reagent for the cell viability assay, 3-(4,5-dimethylthiazol-2-yl)-5-(3-carboxymethoxyphenyl)-2-(4-sulfophenyl)-2H-tetrasolium (MTS) (Promega Corp, Madison, WI) was stored at −20°C until thawed for each use. The colorimetric absorption was performed on a 96-well plate reader (Synergy Mx; Biotek Instruments, Inc., Winooski, VT).

### Cell Culture

PC12 cells (American Type Culture Collection, Manassas, VA) were plated and grown until 80% confluent in proliferation media composed of Dulbecco's modified Eagle's media (DMEM) supplemented with 12.5% horse serum and 2.5% fetal bovine serum. Cells were then scraped, centrifuged, and resuspended in fresh proliferation media using a 20-gauge needle for trituration. Cell concentration was quantified using and automated cell counter (Countess Cell Counter; Invitrogen, Carlsbad, CA). Cells were then carried into a 96-well flat bottom plate at a density of 1.5 × 10^4^ cells/mL. We did not carry cells into the wells along the perimeter of the plate to avoid differential growth conditions that can be seen on these wells. Cells were then grown to 70% confluence, at which time growth media were replaced with differentiation media composed of DMEM supplemented with 1% horse serum. All media contained 1% (wt/vol) penicillin-streptomycin (Invitrogen). All cells were grown in 37°C incubators at 5% ambient levels of carbon dioxide. All media were replaced every other day.

For cell viability experiments, drugs were diluted to the appropriate concentration in DMEM cell media. We exposed cells to drug conditions for predetermined durations. Controls were incubated with DMEM only (“live” in equation below) or 50% ethanol in DMEM for 30 minutes (“dead” in equation below). Each condition (drug concentration and duration of exposure) including controls were performed in *N* = 18 replicate wells. Colorimetric absorbance at 490 nm light was measured after 30, 60, 120, and 240 minutes using the published protocol for the MTS colorimetric assay.^15^ The mean average absorbance values (abs) for each condition (*x*) were then converted into percentage viability by subtracting the mean average absorbance of our “dead” cell control, divided by the “live” cell control, and multiplied by 100.

$$\newcommand{\bialpha}{\boldsymbol{\alpha}}\newcommand{\bibeta}{\boldsymbol{\beta}}\newcommand{\bigamma}{\boldsymbol{\gamma}}\newcommand{\bidelta}{\boldsymbol{\delta}}\newcommand{\bivarepsilon}{\boldsymbol{\varepsilon}}\newcommand{\bizeta}{\boldsymbol{\zeta}}\newcommand{\bieta}{\boldsymbol{\eta}}\newcommand{\bitheta}{\boldsymbol{\theta}}\newcommand{\biiota}{\boldsymbol{\iota}}\newcommand{\bikappa}{\boldsymbol{\kappa}}\newcommand{\bilambda}{\boldsymbol{\lambda}}\newcommand{\bimu}{\boldsymbol{\mu}}\newcommand{\binu}{\boldsymbol{\nu}}\newcommand{\bixi}{\boldsymbol{\xi}}\newcommand{\biomicron}{\boldsymbol{\micron}}\newcommand{\bipi}{\boldsymbol{\pi}}\newcommand{\birho}{\boldsymbol{\rho}}\newcommand{\bisigma}{\boldsymbol{\sigma}}\newcommand{\bitau}{\boldsymbol{\tau}}\newcommand{\biupsilon}{\boldsymbol{\upsilon}}\newcommand{\biphi}{\boldsymbol{\phi}}\newcommand{\bichi}{\boldsymbol{\chi}}\newcommand{\bipsi}{\boldsymbol{\psi}}\newcommand{\biomega}{\boldsymbol{\omega}}\% {\rm{viability}}\left( x \right) = \left( {{{\sum {\rm{abs}}\left( x \right)} \over {18}} - {{\sum {\rm{abs}}\left( {{\rm{dead}}} \right)} \over {18}}} \right)\left/ \left( {{{\sum {\rm{abs}}\left( {{\rm{live}}} \right)} \over {18}}} \right) \times 100\right.$$

### Animals

Male Sprague-Dawley rats (Charles River Laboratories, Wilmington, MA) were housed in a 6 AM to 6 PM light and dark cycle. Animals were cared for in accordance with protocols approved by the Animal Care and Use Committee at Boston Children's Hospital (Boston, MA). Animal care conformed to the Guide for the Care and Use of Laboratory Animals of the US National Research Council and the ARVO statements for the Use of Animals in Ophthalmic and Vision Research.^16^

### Topical Corneal Drug Administration and Nociceptive Testing

Using a method previously published by our laboratory,^10^,^17^ rats were gently towel restrained with the eyes held open, and 30 μL of a drug solution was applied to one eye. After 15 seconds, the eye was gently closed to spread drug over the cornea. Every 15 minutes after drug administration, the eye was probed with a Cochet-Bonnet esthesiometer filament (Luneau Ophthalmologie, Chartres, France) with the animal under gentle towel restraint. This filament is adjustable between 6 and 0.5 cm, with shorter filament lengths providing a more noxious stimulus.^18^ Starting at 6-cm filament length and decreasing by 0.5-cm increments, the eye was probed three times at each length until a blink response was elicited. The length recorded for each time point was the shortest length of the monofilament where blink response was not present. Testing was discontinued in a given rat when there was a blink response present at a monofilament length of 6 cm. The operator was blinded to drug and drug concentration.

Nociceptive blockade was categorized into complete for rats that had no blink response at the shortest filament length of 0.5 cm (Block_0.5_), deep for rats that had no blink response with the monofilament length from 1 to 3 cm (Block_3_), and partial for rats that had no blink response with the monofilament length from 3.5 to 6 cm (Block_<6_).

### Repeated Topical Drug Administration on Intact Corneas

Rats were gently towel restrained and received 30 μL of either 15 or 100 mM QX-314 concentration on one eye. On the contralateral eye we applied either vehicle (0.9% NaCl) or 15 mM PPC in a similar fashion (*n* = 5 eyes per condition with four total conditions, total of 10 animals). This was done hourly for 12 hours. Animals were then euthanized with carbon dioxide. Five additional animals who had received no treatments were also euthanized to serve as negative controls. Intact whole eyes were harvested for histologic analysis and fixed in 10% neutral buffered formalin. Hematoxylin and eosin staining and periodic acid-Schiff staining were performed on paraffin-embedded corneal sections and were evaluated qualitatively for evidence of epithelial, stromal, and endothelial changes. Histologic examination was performed by a board certified pathologist (MM) who is fellowship trained in ophthalmic pathology and was blinded to drug and drug concentration.

### Corneal Epithelial Reepithelialization

Rats were anesthetized with isoflurane (Baxter International, Inc., Deerfield, IL) and then were removed from isoflurane and received an intraperitoneal injection of 50 mg/kg ketamine (Hospira, Inc., Lake Forest, IL) and 10 mg/kg xylazine (Lloyd, Inc., Shenandoah, IA). After rats were fully anesthetized, under a dissecting microscope and sterile conditions a 3-mm biopsy punch (Miltex, Inc., York, PA) was used to create a unilateral circular corneal epithelial defect. A corneal rust ring remover (Algerbrush II; Alger Company, Inc., Lago Vista, TX) with a 1-mm burr tip was used to debride the corneal epithelium from within the borders of the defect. Care was taken to make a uniform circular defect without epithelial remnants. Immediately following epithelial debridement, 30 μL of QX-314 or PPC were administered on the eye. Rats also received a single dose of 0.1 mg/kg buprenorphine (Reckitt Benckiser, Slough, UK) injected subcutaneously for postoperative pain control. After the first topical instillation of drug, 30 μL of each drug concentration was reinstilled into the eye at intervals equal to the time to resolution of Block_3_. PPC and 15 mM QX-314 were therefore instilled every 1 hour and 50 and 100 mM QX-314 every 2 hours until complete closure of the corneal defect, typically less than 36 hours.

To visualize the defect, sterile 0.6 mg fluorescein sodium ophthalmic strips were placed in 100 μL phosphate-buffered saline. The resulting fluorescein sodium solution (15 μL) was then instilled onto the eye immediately following defect creation and every 8 hours during the first 24 hours, then every 4 hours from 24 hours until epithelial defect closure. Photographs of the eye were taken at each time point (D90; Nikon, Tokyo, Japan) with a 40-mm f/2.8 lens (AF-S DX Micro NIKKOR; Nikon) under cobalt blue light provided by an ophthalmoscope (Welch Allyn, Inc., Skaneateles Falls, NY). The two-dimensional area of the initial defect was measured using ImageJ software (imagej.nih.gov/ij/; provided in the public domain by the National Institutes of Health, Bethesda, MD). We then calculated the corneal reepithelialization rate as the area of the initial defect divided by time to complete defect healing. The operator creating the corneal defect and making measurements was masked to drug and drug concentration.

### Statistical Analysis

Data are reported as means and standard deviations of *N* observations. Comparisons across multiple concentrations of QX-314 and PPC block durations were performed using 1-way ANOVA as in the Table. Throughout the article, comparisons of duration of anesthesia, depth of anesthesia, cell viability, and corneal reepithelialization rates between individual drug concentrations are performed using 1-way ANOVA. In cases where there were multiple comparisons, Bonferroni correction was used, in which the *P* value from ANOVA was multiplied by the number of comparisons performed, and a two-sided α value was kept at 0.05. *P* values for corneal anesthesia were corrected for four comparisons (four concentrations of QX-314 compared to PPC). Cell viability *P* values were corrected for three comparisons (three concentrations of QX-314 compared to PPC at 30 minutes). Corneal reepithelialization analysis was corrected for five comparisons (four concentrations of QX-314 and 0.9% saline compared to PPC). Fischer's exact test was used to determine *P* values for comparisons of proportions of animals achieving complete block. All statistical analyses were performed using statistical software (GraphPad Prism version 7.0; GraphPad Software, Inc., La Jolla, CA).

**Table:** Block Durations, Initial Depth, and Corneal Reepithelialization Rates After Drug Exposure

Concentration	Duration of Block_<6_, min	Duration of Block_3_, min	Duration of Block_0.5_, min	Block Depth 15 Min, cm	Animals Achieving Complete Block	Reepithelialization Rate, mm^2^/h
PPC, 15 mM	87 ± 16	54 ± 17	0 ± 0	2 ± 0.8	0/5	0.25 ± 0.03
QX-314, 15 mM	114 ± 17	66 ± 23	0 ± 0	1.8 ± 0.3	0/5	0.39 ± 0.10
QX-314, 25 mM	135 ± 15	90 ± 24	9 ± 13	1.2 ± 0.8	2/5	Not tested
QX-314, 50 mM	180 ± 18	114 ± 13	12 ± 16	0.9 ± 0.4	2/5	0.37 ± 0.08
QX-314, 100 mM	174 ± 13	132 ± 16	15 ± 15	0.7 ± 0.3	3/5	0.38 ± 0.04
0.9% Saline	0 ± 0	N/A	N/A	N/A	N/A	0.44 ± 0.06
*P* value	<0.0001	<0.0001	0.17	0.007	N/A	0.012

Block_<6_, partial corneal anesthesia; Block_3_, deep corneal anesthesia; Block_0.5_, complete corneal anesthesia.

## Results

### Corneal Anesthesia

**Figure 1:**
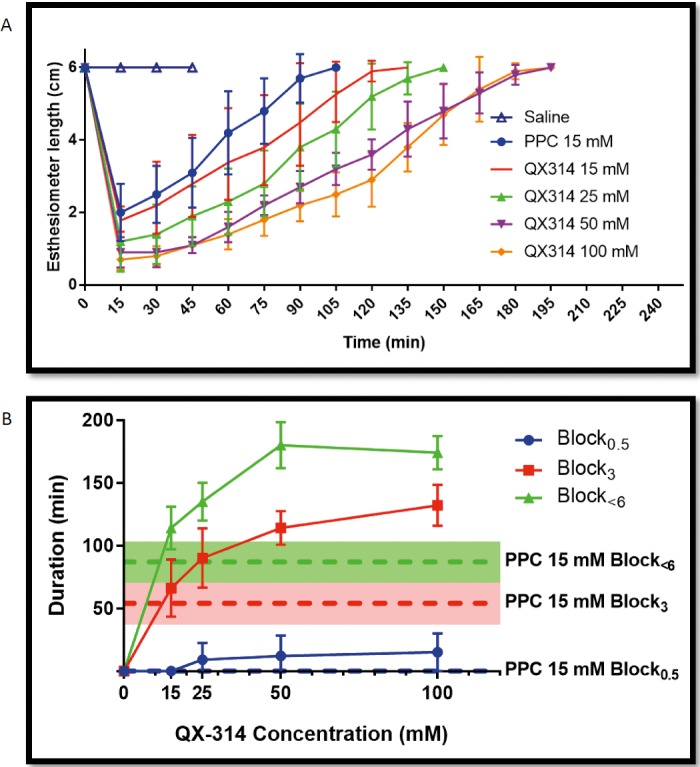
(A) Time course of corneal anesthesia. The y-axis shows the filament length on a Cochet-Bonnet esthesiometer needed to elicit a blink after single application of topical anesthetics. (B) Durations of Block_0.5_ (complete), Block_3_ (deep), and Block_<6_ (partial) corneal anesthesia following a single topical application of corneal anesthetics. Data are means ± SD (N = 5) and were calculated from the data presented in (A). No animals in the 15 mM PPC or 15 mM QX-314 groups achieved Block_0.5_.

To test the hypothesis that QX-314 would provide prolonged duration corneal anesthesia, 30 μL of either 15 mM PPC ophthalmic solution, or 15, 25, 50, or 100 mM concentrations of QX-314 were administered once topically to the corneal surface in rats (*n* = 5 per drug concentration). A Cochet-Bonnet esthesiometer was then used to determine corneal sensitivity at predetermined time points.^18^ (In Fig. 1A, note that the top of the *y*-axis [6 sec] represents recovery to normal sensation from local anesthesia.) The durations of topical anesthesia from each test formulation were then calculated as in Materials and Methods (Fig. 1B, Table).

Animals treated with 15 mM QX-314 had a similar partial block duration (Block_<6_), where anesthesia is partial or better (i.e., it is also the total duration of nerve block) compared with PPC 15 mM ophthalmic solution (114 ± 17 and 87 ± 16 minutes, respectively; *P* = 0.06). The duration of anesthesia increased with increasing concentration of QX-314, then did not increase further at 100 mM. The durations of Block_<6_ at 50 and 100 mM, 180 ± 18 and 174 ± 13 minutes, respectively, were approximately twice as long as that achieved with 15 mM PPC, 87 ± 16 (*P* < 0.0001). The duration of deep block (Block_3_) was similar between 15 mM PPC, 54 ± 7.6 minutes, and 15 mM QX-314, 66 ± 10 minutes (*P* = 0.95), but 2.4-fold shorter than that from 100 mM QX-314, 132 ± 7 minutes (*P* < 0.0001). There were no statistically significant differences in the duration of complete block (Block_0.5_) between groups (*P* = 0.17). However, the average depth of block at the first time point after drop application (15 minutes) was significantly different across groups (Table), with significantly stronger stimuli needed to elicit a blink in higher concentrations of QX-314 compared with PPC (*P* = 0.007). Three out of five animals receiving 100 mM QX-314 achieved complete block (no blink with a 0.5-cm filament length), while none of the animals treated with PPC or 15 mM QX-314 achieved complete block. However, this difference was not statistically significant (Fischer's exact, *P* = 0.17).

### Cell Viability

**Figure 2:**
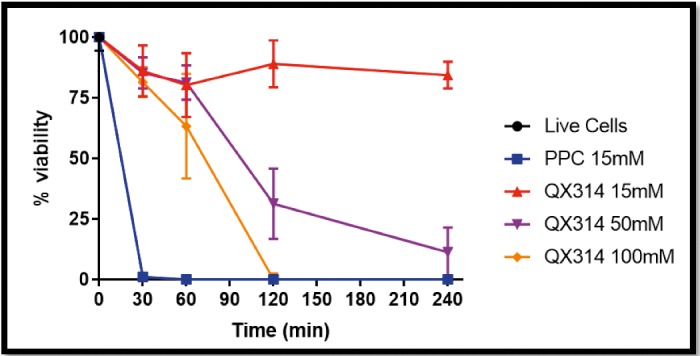
Effect of drug, concentration, and duration of exposure on PC12 cell viability using the MTS cell viability assay. Data are means ± SD.

To test the hypothesis that QX-314 would have minimal toxicity, we exposed differentiated cells from the PC12 cell line to 15 mM PPC and various concentrations of QX-314 (*n* = 18 per drug concentration and duration of exposure) (Fig. 2). PC12 cells are a pheochromocytoma cell line that can be used in in vitro evaluations of neurotoxicity.^19^ MTS cell viability assays were performed at predetermined time points (using untreated cells and ethanol-killed cells to establish bounds for 100% and 0% viability, respectively; see Materials and Methods). Cells treated with 15 mM QX-314 for 30 minutes demonstrated 86.1% ± 10.5% viability compared with 1.1% ± 0.5% in cells treated with PPC 15 mM (*P* < 0.0001). Cellular viability remained 84.4% ± 5.5% after 240 minutes of continuous exposure to 15 mM QX-314. Higher concentrations of QX-314 showed decreasing cellular viability over time. However, even 100 mM QX-314 had good cell viability at 30 and 60 minutes (84.9% ± 6.1% and 67.1% ± 21.3%, respectively).

### Corneal Histology

**Figure 3:**
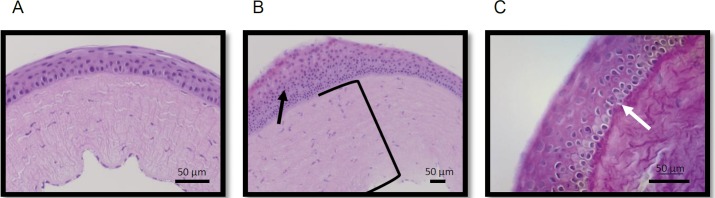
Representative corneal specimens from animals with intact corneas treated hourly with 100 mM QX-314 for 12 hours. Histologic findings demonstrated in these images were seen at similar frequencies in animals treated with saline, PPC, and all tested concentrations of QX-314. (A) Central corneal hematoxylin and eosin (H+E) staining showing normal corneal architecture. (B) Central corneal H+E staining showing stromal edema (black bracket) with focal increased epithelial proliferation (black arrow). (C) Central corneal periodic acid-Schiff staining showing minimal basal layer changes/vacuolar effects (white arrow).

To test the hypothesis that QX-314 would have minimal in vivo toxicity on intact corneal epithelia, 30 μL sterile saline, 15 mM PPC ophthalmic solution, low-dose (15 mM) or high-dose (100 mM) QX-314 were administered topically every hour for 12 hours to the intact corneal surfaces of rats. Histologic examination of the corneas after necropsy demonstrated qualitatively similar findings in all groups, including five untreated animals (Fig. 3). Nonspecific findings such as focal areas of stromal edema and minimal basal layer change/vacuolar effect were observed in all groups, including saline-treated and untreated controls. These findings were therefore not attributable to specific treatment groups but rather might have been due to minor corneal traumatic injuries in the setting of communal housing of the animals. There were no instances of ulceration or keratitis in any of the groups.

### Corneal Defect Healing

**Figure 4:**
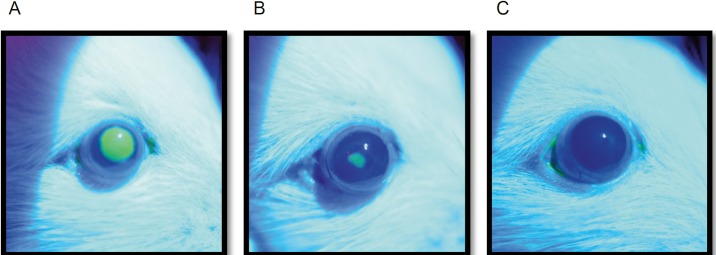
Corneal reepithelialization after debridement. Representative fluorescent images obtained at (A) the time of defect creation, (B) after 24 hours, and (C) after 36 hours.

**Figure 5:**
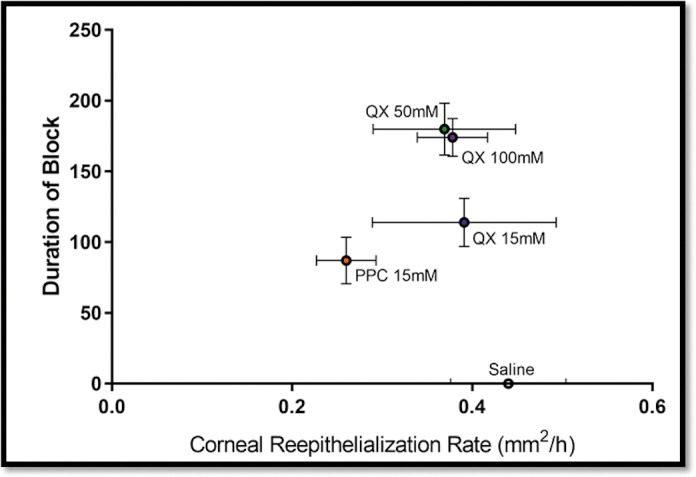
Relationship between the rate of corneal reepithelialization and the duration of Block_<6_ (i.e., partial or greater block) after repeated dosing of topical anesthetics. Data are means ± SDs. N = 4 rats per group.

We then studied the effect of QX-314 on corneal epithelial healing rates. PPC has previously been shown to delay corneal healing.^7^,^17^ We applied sterile saline, PPC, or various concentrations of QX-314 to the eye of rats with a unilateral experimental corneal defect, visualized with fluorescein (Fig. 4) (*n* = 4 per group). We used corneal healing rates (mm^2^/h) rather than time to complete healing to account for minor (<1 mm^2^) differences in initial defect size. Corneal healing rates are shown in the last column of the Table and in Figure 5, along with the time until the block resolved completely (Block_<6_).

The healing rate with 15 mM PPC was 0.25 ± 0.03 mm^2^/h, significantly slower than that with 0.9% saline, 0.44 ± 0.06 mm^2^/h (*P* = 0.003), and 15 mM QX-314, 0.39 ± 0.10 mm^2^/h (*P* = 0.04). The healing rate in animals treated with 15 mM PPC was not statistically significantly different from those with 50 mM QX-314 (0.37 ± 0.08 mm^2^/h) and 100 mM QX-314 (0.38 ± 0.04 mm^2^/h), (*P* = 0.10 and *P* = 0.07, respectively, by 1-way ANOVA after Bonferroni correction for five total comparisons; see additional analysis below). We note, however, that if the corneal reepithelialization data from the 50- and 100-mM QX-314 groups (which had very similar durations of corneal anesthesia and rates of reepithelialization) are pooled in a single high-concentration QX-314 group, their rate of reepithelialization was statistically significantly faster (0.37 ± 0.06 mm^2^/h, *n* = 8) compared to 15 mM PPC (0.25 ± 0.03 mm^2^/h, *n* = 4) (*P* = 0.04 after Bonferroni correction for five total comparisons). Additionally, the corneal healing rate for 15, 50, and 100 mM QX-314 were not statistically different from those for saline (1-way ANOVA *P* values = 0.94, 0.46, and 0.62).

## Discussion

QX-314 provided safe and prolonged duration corneal anesthesia in a rodent model. Increasing concentrations were associated with improved duration and depth of anesthesia. We compared this with PPC, in a formulation and concentration at which it is used clinically as a topical ocular anesthetic, 0.5% (15 mM). The depth and duration of corneal anesthesia achieved with 15 mM QX-314 was similar to equimolar PPC. All tested concentrations ranging from 25 to 100 mM QX-314 offered superior durations and depths of corneal anesthesia than did 15 mM PPC. The highest concentration of 100 mM QX-314 was significantly more effective at achieving complete block 15 minutes after application than was PPC or lower concentrations of QX-314. All concentrations of QX-314 also demonstrated less cytotoxicity to PC12 cells than did 15 mM PPC. In vivo, there were no major corneal histologic changes associated with short-term repeated use of QX-314. Furthermore, for a similar depth and duration of nociceptive block, equimolar (15 mM) doses of QX-314 caused less delay of corneal epithelial healing than did PPC. Individual comparisons of PPC 15 mM with the higher concentrations of QX314 (50 and 100 mM) had statistically similar rates of corneal reepithelialization. A post hoc analysis was performed in which pooled reepithelialization rates of 50 and 100 mM QX-314–treated animals were significantly faster than those receiving PPC 15 mM. Overall, QX-314 at these concentrations did not appear to exert significant inhibition on corneal healing. Taken together, these data suggest a plausible role for QX-314 as a topical corneal anesthetic in humans.

Conventional LAs have been successfully used topically to facilitate ophthalmic surgery; however, their duration of action is typically short, and their application may delay corneal epithelial healing.^7^,^17^ Our in vitro data also demonstrate significant toxicity of PPC on a neuroblastic cell line. Approaches including subconjunctival and regional injection techniques (peribulbar and retrobulbar routes) have been used to overcome these limitations.^20^ Regional anesthetic techniques can provide longer duration of block and globe akinesia but carry rare but serious risks of sight-threatening retinal or optic nerve injury, globe penetration, conjunctival edema, lid and retrobulbar hemorrhage, seizure, and cardiac arrhythmia.^21^ This study indicates that QX-314 may be a useful addition to the armamentarium of topical ophthalmic anesthetics available to the ophthalmologist, an agent with prolonged duration of anesthesia and minimal toxicity. Potential uses of topical ophthalmic QX-314 include facilitation of corneal surgery requiring prolonged, deep corneal anesthesia without the need for globe akinesia.

The mechanism by which QX-314 causes prolonged duration ophthalmic anesthesia is not clear. Previous studies of perineural injection of quaternary lidocaine derivatives at the sciatic nerve demonstrated prolonged nerve block compared to conventional LAs.^12^,^13^ The permanent cationic charge of QX-314, which inhibits the penetration of lipid membranes^22^ to the drug's site of action on the cytoplasmic side of the sodium channel,^23^,^24^ is believed to result in slow onset of nerve block and may also contribute to the prolonged effect. Finally, the mechanism of how the charged QX-314 might penetrate the corneal epithelial tight junctions to reach the sensory nerve fibers remains unclear.

While charged molecules have poor penetration of biological membranes, it is thought that QX-314 can enter sensory nerves through cation channels, specifically the transient receptor potential vanilloid family of cation channels including TRPV1,^11^,^25^ which is present in corneal sensory nerves.^26^–^30^ This channel opens in response to thermal stimuli, certain irritants, and noxious mechanical stimuli. It is also suggested that at higher concentrations, QX-314 may simply diffuse across the axonal membrane, providing nerve block.^25^ Future studies should investigate the role of TRPV1 and other vanilloid receptor agonists on QX-314 anesthesia. Furthermore, the LA effects of QX-314, like other conventional LAs, is known to be enhanced by adjuncts, including chemical permeation enhancers, dexmedetomidine, and site 1 sodium channel blockers.^17^,^31^–^33^ It remains to be seen whether coadministration of such adjuncts with QX-314 would improve topical corneal anesthesia.

The effectiveness and risks of repeated application of QX-314 over longer time frames, as would be used for the treatment of chronic eye pain, remain unclear. Conventional LAs are believed to entail the risk of corneal ulceration and toxic keratopathy, sometimes referred to as neurotrophic keratopathy, with prolonged or excessive use.^34^,^35^ There is currently no approved long-acting topical LA treatment for chronic eye pain. Further study is required to determine whether QX-314—or agents like it—would be suitable for treating chronic pain.

Finally, it remains unclear whether the current formulation in 0.9% saline is optimal. It is possible that at high concentrations of QX-314, hypertonicity of the solution could lead to discomfort and reflex tearing. QX-314 at concentrations up to 100 mM are soluble in water at room temperature.^36^ Future work can investigate the role of the diluent, pH adjustment, and buffering on the efficacy and tolerability of topical ophthalmic QX-314 administration.

Our study compared the safety and efficacy of the cationic lidocaine derivative, QX-314, over concentrations from 15 to 100 mM for topical ophthalmic anesthesia. We tested its anesthetic characteristics and toxicity profile in reference to a commonly used topical ophthalmic LA, 15 mM (0.5%) PPC. Our data suggest that QX-314 may provide safe, prolonged duration topical ophthalmic anesthesia.
